# Application of Amino Acid-Based Carbon Dots for the Treatment of Oral Bacteria and Oral Cancer Cells In Vitro Using a Dental Light-Curing Unit via ROS-Mediated Therapy

**DOI:** 10.3390/nano15211677

**Published:** 2025-11-05

**Authors:** So-Young Park, Wooil Kim, Unchul Shin, Yong Hoon Kwon, Franklin Garcia-Godoy, Hye-Ock Jang

**Affiliations:** 1 Department of Pediatric Dentistry, Dental and Life Science Institute, School of Dentistry, Pusan National University, Yangsan 50612, Republic of Korea; syparkpedo@pusan.ac.kr; 2 Department of Dental Materials, Dental and Life Science Institute, School of Dentistry, Pusan National University, Yangsan 50612, Republic of Korea; kimoo1@pusan.ac.kr (W.K.); y0k0916@pusan.ac.kr (Y.H.K.); 3 Department of Bioscience Research, College of Dentistry, University of Tennessee Health Science Center, Memphis, TN 38103, USA; 4 Department of Dental Pharmacology, Dental and Life Science Institute, School of Dentistry, Pusan National University, Yangsan 50612, Republic of Korea

**Keywords:** carbon dots, oral bacteria, oral cancer, dental light-curing unit, dentistry

## Abstract

In systemic diseases, controlling oral bacteria and cancer is an important issue. As biomaterials, recently, carbon dots (DSs) are the focus of a variety of studies owing to their extensive applicability in life sciences. In this study, the effectiveness of carbon dots (CDs) for the elimination of both oral bacteria and oral cancer in vitro was assessed using a dental light-curing unit (LCU) as a light source. CDs were synthesized using an amino acid. The absorbance of CDs and the emission spectrum of the LCU were measured. The production of reactive oxygen species (ROS) was evaluated spectroscopically. Changes in glutathione (GSH) content were evaluated. Using oral bacteria and cancer cells, in vitro antibacterial and antitumor capabilities of CDs were evaluated under light irradiation. Confocal microscopy was used to observe live/dead cells and intracellular lipid peroxidation (LPO). The emission spectrum of the LCU fully matched the absorbance of CDs. After CD treatment, the initial peak absorbances of the p-nitrosodimethylaniline-imidazole (for singlet oxygen assay) and nitroblue tetrazolium (for superoxide oxide assay) solutions changed under light irradiation. The initial peak absorbance of the GSH assay solution decreased during and after light irradiation. Both CD-treated oral bacteria and oral cancer cells were near totally eliminated at 50 and 200 μg/mL concentrations, respectively, after light irradiation. In the live/dead cell and C11-BODIPY^581/591^ dye assays, red and green fluorescent spots were, respectively, observed in the CD-treated and light-irradiated cells. Accordingly, CDs effectively eliminated both oral bacteria and cancer cells in vitro in conjunction with dental LCU with less damage to normal cells through ROS-induced or ROS-initiated GSH depletion-induced intracellular LPO. Dental LCU plays a crucial role in ROS production through CD photoexcitation. Dental LUC has the potential to be used as a light source in dentistry for the treatment of oral bacteria and cancer cells.

## 1. Introduction

The oral cavity, although not physically large, is structurally complex and has multiple anatomical components and diverse oral microbiota. There are more than 20 billion oral pathogens, comprising approximately 700 predominant taxa [1,2]. In a healthy mouth, despite the presence of diverse microbial communities, oral pathogens maintain an equilibrium until this balance is damaged. Some bacterial species are harmful to oral health and are associated with various oral diseases.

As to oral diseases, caries, produced by acidogenic *Streptococcus mutans* (*S. mutans*), is one of the most common [3,4]. *Enterococcus faecalis* (*E. faecalis*) is frequently found in treated root canals and is implicated in treatment failure, which may require retreatment owing to incomplete or inadequate treatment [5,6]. A commensal fungus *Candida albicans* (*C. albicans*) can cause candidiasis (thrush) if it overgrows and the immune system weakens [7,8]. Oral squamous cell carcinoma occurs in the oral epithelium, with approximately 400,000 cases and 45% mortality by various carcinogenic risk factors, such as smoking and alcohol consumption [9].

Recently, the perspective that oral bacteria affect the incidence of whole-body diseases (systemic diseases), such as cancer and vascular and neurological diseases, has attracted great attention owing to the findings of extensive meta-analyses [10,11]. As a disease source, oral bacteria inhabiting the oral cavity are significant in the incidence of systemic diseases. Furthermore, oral cancer is highly likely to interact with oral bacteria in the oral cavity; any simple and economical treatment that can prevent this interaction and be directly applied at the chairside would be highly valuable.

Among studies on the treatment of bacteria and cancers, the outcomes achieved using carbon dots (CDs) have been extraordinary. Forming an extensive family of carbon allotropes, CDs exhibit many valuable optical, chemical, and electrochemical characteristics that can extend their applications to many fields [12,13]. Among the features of CDs, the production of reactive oxygen species (ROS) extends their feasibility of CDs to the treatment of bacteria and cancers. CDs can be easily adopted and applied because they can be synthesized using inexpensive resources with a simple heat treatment. Additionally, their workability can be further extended by modifying their structures and adding diverse functionalities. Among carbon sources, amino acids are attractive because they contain both amino and carboxyl groups, which act as active sites on the CD surface to enhance water solubility, antibacterial activity, and binding ability to other molecules [14]. Methionine is a sulfur-containing essential amino acid that plays a vital role in the production of antioxidants (e.g., glutathione, cystathionine) and other non-essential amino acids. Owing to specific fluorescence response and potential biomedical applications of methionine-based CDs, methionine can be a useful precursor for CDs [15,16]. When applying CDs, additional light irradiation enhances the reaction outcomes owing to the incidence of various light-induced effects, such as photodynamic and photocatalytic effects. In dentistry, light irradiation is the basic procedure used for dental restorations using resin composites with an aid of blue light [17]. LED-based blue light-emitting light-curing units (LCUs) are the basic equipment in dental clinics. However, except for such usage, using LED as a light source for light-induced effects is rare [18]. Under these circumstances, treatment of oral bacteria and cancer using CDs in conjunction with LCUs can be challenging for the confrontation of systemic diseases in dentistry. In this study, we aimed to evaluate whether the synthesized CDs could eliminate both oral bacteria and cancer in vitro by using a LCU as a light source. Through these experimental studies, the possible antibacterial and antitumor mechanism was explored.

## 2. Materials and Methods

### 2.1. Synthesis of CDs

CDs were prepared for this study. Briefly, 100 mg of o-phenylenediamine was dissolved in 10 mL of ethanol; 7 mL of deionized water containing 140 mg of methionine was added. After stirring for 10 min, the mixture was transferred into a Teflon-lined stainless-steel autoclave, sealed, and heated to 180 °C for 16 h in a furnace. After cooling overnight, the supernatant was collected by centrifugation, rinsed, and dried in an oven at 60 °C overnight. The dried powder (CDOM) was stored in a refrigerator until use. All the used reagents were purchased from Sigma-Aldrich (St. Louis, MO, USA) otherwise specified.

### 2.2. Characterization of CDOM

The absorbance of CDOM and emission spectrum of LCU (Bluephase, Ivocla Vivadent, Schaan, Liechtenstein) was measured using a microplate reader (BioTek Synergy HTX; Agilent, Santa Clara, CA, USA) and UV-VIS spectrometer (HR4000, Ocean Optics Inc., Dunedin, FL, USA), respectively. The fluorescence emitted from CDOM by LCU excitation was obtained using a spectrofluorometer (QE65000FL, Ocean Optics Inc., Dunedin, FL, USA). The nanomorphology of CDOM was observed using TEM (Talos F200X, Thermo Fisher Scientific, Waltham, MA, USA). FTIR spectra were recorded using FTIR Spectrometer (Thermo Fisher Scientific). XPS analysis was done using XPS spectrometer (K-ALPHA XPS, Thermo Fisher Scientific).

### 2.3. Evaluation of ROS Production

To evaluate the production of singlet oxygen (^1^O_2_), a p-nitrosodimethylaniline-imidazole (RNO-ID) assay was performed. To prepare the stock solution, 0.23 mg of RNO and 16.35 mg of ID were mixed in 50 mL of deionized water and stirred for 10 min. Thereafter, 200 μL of the prepared stock solution was mixed with 100 μM H_2_O_2_ and 100 μL of CDOM solution (200 μg/mL); the mixture was then irradiated using the LCU at 100 mW/cm^2^ intensity for different irradiation times. Optical density (OD) of the test solutions was measured using a spectrophotometer (SpectraMax 190, Molecular Devices, San Jose, CA, USA) at different irradiation times. A solution without CDOM was used as a control.

To evaluate the production of superoxide anion (^•^O_2_^−^), the nitroblue tetrazolium (NBT) assay was performed. Assay solutions were prepared by adding NBT (0.2 mM), NADH (0.5 mM), 100 μM H_2_O_2_, and CDOM (200 μg/mL) to dimethyl sulfoxide. The prepared solution was irradiated, and the corresponding absorbance was measured using the same spectrophotometer at λ = 530 nm during different irradiation times. A solution without CDOM was used as a control.

### 2.4. Antibacterial Activity Test

The antibacterial properties of CDOM were evaluated using two oral bacteria (*E. faecalis* and *S. mutans*) and one fungus (*C. albicans*) commonly found in the oral cavity. The two oral bacteria were cultivated in brain heart infusion (BHI) broth at 37 °C in a shaker (200 rpm) for 24 h and 48 h, respectively, to fully restore their proliferative and metabolic capacities. The bacterial count was adjusted to 2 × 10^6^ CFU/mL at the beginning of the experiment. Specifically, 200 μL of the above bacterial suspensions and 200 μL of CDOM (0, 10, 20, 30, 40, and 50 μg/mL) were co-cultured at 37 °C for 10 min in a 48-well pate. Each well was divided into two groups: no light irradiation and light irradiation at 100 mW/cm^2^ intensity for 4 min. The number of viable bacteria in each group was quantified using a standard continuous dilution method. Finally, 100 μL of the culture suspensions were evenly spread on the corresponding agar plates, and the number of colonies was counted after culturing *E. faecalis* for 24 h and *S. mutans* for 48 h. The bacterial viability (%) was calculated as follows:

(1) Bacterial viability (%) = (N_control_ − N_sample_)/N_control_ × 100%.

*C. albicans* were cultivated in yeast extract peptone dextrose (YPD) broth at 37 °C for 24 h. The fungal count was adjusted to 2 × 10^5^ CFU/mL at the beginning of the experiment. Specifically, 200 µL of the *C. albicans* suspensions and 200 µL of CDOM (0, 25, 50, 100, 150, and 200 µg/mL) were co-cultured at 37 °C for 10 min in a dish. After dividing specimens into two groups (no light irradiation and light irradiation at 100 mW/cm^2^ intensity for 3 min) and after standard serial dilutions, 50 µL of each culture suspension was evenly spread onto the corresponding agar plates. Colonies were counted manually after incubating *C. albicans* in a dish for 24 h.

### 2.5. Cell Viability Test

Two cell lines (HSC3 and HEK293) were used in this study. The human tongue squamous carcinoma cell line (HSC3) was obtained from the Japanese Cancer Research Resources Bank and cultured in Dulbecco’s modified Eagle’s medium (DMEM) F12 supplemented with 10% heat-inactivated fetal bovine serum and 1% penicillin/streptomycin. The human embryonic kidney cell line (HEK293) was obtained from the Korean Cell Line Bank and cultured in DMEM. After culturing, all cells were maintained in a 5% CO_2_ atmosphere at 37 °C.

To assess cell viability, cells were seeded in 96-well plates (1 × 10^4^ cells/well) and incubated for 12 h before treatment. The cells were then treated with CDOM solution at the desired concentrations for 24 h (HEK293 normal cells, without light irradiation). HSC3 cells were treated with CDOM at different concentrations and 100 μM H_2_O_2_ without or with 0.1 μg/mL ferrostatin-1 (Fer-1) and were incubated for 4 h, without or with light irradiation using the LCU for 30 s at 50 mW/cm^2^ intensity; they were additionally incubated for 20 h. The culture medium was replaced with fresh medium containing 10% CCK-8 solution (DOJINDO, Kumamoto, Japan), and the cells were incubated for 1 h. The absorbance of the cells in each well was measured at 450 nm using a microplate reader (BioTek Synergy HTX).

After cell-viability tests, the cells were observed under an optical microscope (Nikon; Eclipse Ts2, Tokyo, Japan).

### 2.6. Evaluation of GSH Depletion

To evaluate the change in GSH content in the test solution, GSH (1 mM) was mixed with CDOM (200 μg/mL) and 100 μM H_2_O_2_, then subsequently, mixed with 100 μM DTNB and light irradiated for 3 min. After the termination of light irradiation, the absorbance at 410 nm was measured at different time points using the same microplate reader.

### 2.7. Live/Dead Cell Staining Assay

Results of cell-viability tests were noted after live/dead cell staining. Briefly, cells were seeded in 96-well plates (1 × 10^4^ cells/well) and incubated for 12 h. Each 500 μL cell suspension (1 × 10^5^ cells/mL) was treated with CDOM at the desired concentration in 100 μM H_2_O_2_ solution, incubated for 4 h, light-irradiated using the LCU for 3 min at 100 mW/cm^2^ intensity, and incubated for 20 h. The cells were washed with phosphate-buffered saline (PBS) several times and stained with Calcein-AM/PI (propidium iodide) dye for 20 min (5 μM Calcein-AM, 50 μg/mL PI). After incubation for 20 min in the dark, cells were observed under a confocal microscope (LSM700, Carl Zeiss, Jena, Germany). In the microscopic images, live and dead cells appeared green and red, respectively.

### 2.8. Intracellular LPO Detection

To evaluate lipid oxidation within cells, intracellular LPO detection was performed. HSC3 cells were seeded evenly in 24-well plates at a density of 5 × 10^4^ cells/well and incubated for 4 h. The cells were then treated with PBS and CDOM and were irradiated with light for 3 min. Cells were stained with C11-BODIPY^581/591^ dye (8 μM; Sigma-Aldrich, St. Louis, MO, USA) for 30 min at 37 °C and washed three times with PBS. Finally, the same confocal microscope was used to obtain fluorescence images of the treated cells.

### 2.9. Statistical Analysis

Results from the bacterial and cell viability tests without and with light irradiation were analyzed using *t*-test. *p*-values < 0.05 were considered significant.

## 3. Results

**Figure 1:**
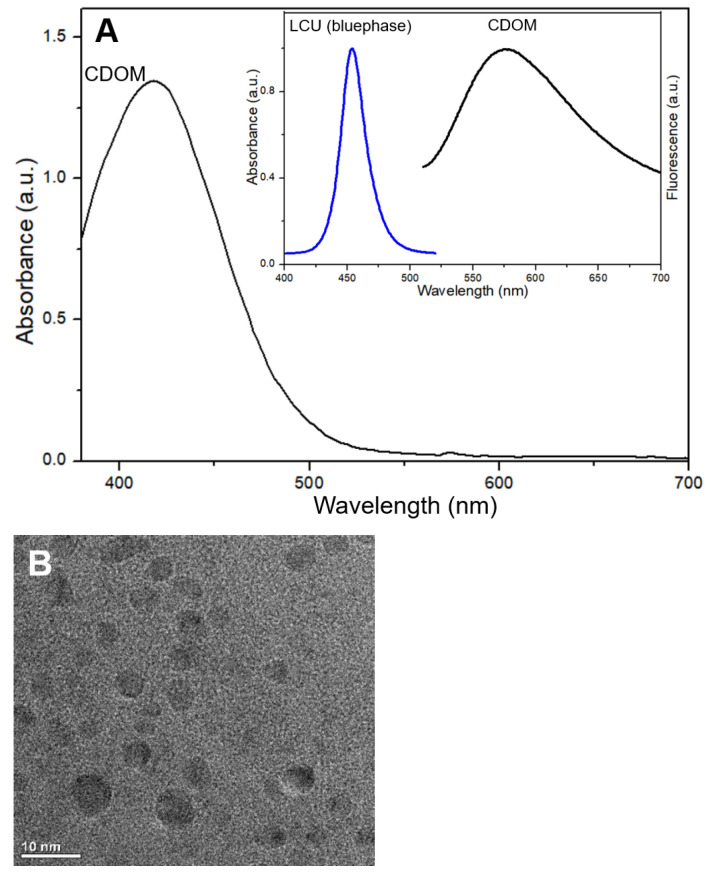

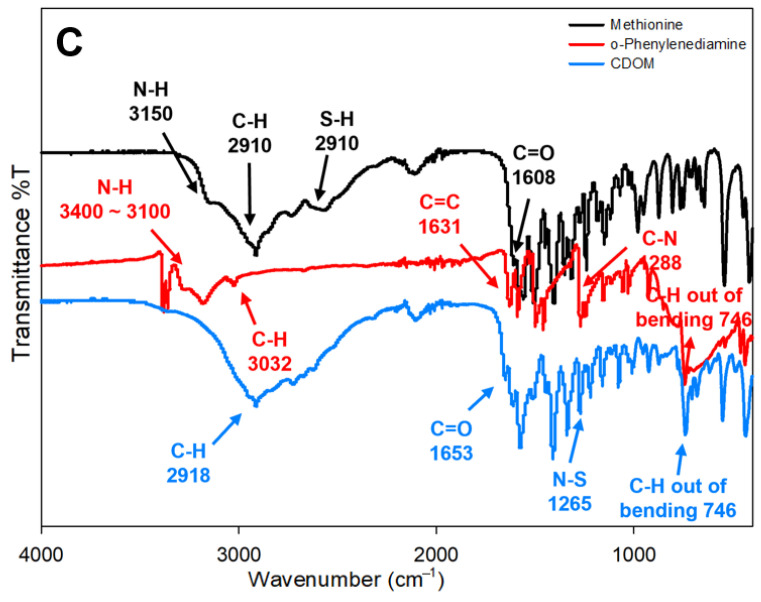
Light absorbance of carbon dots (CDOM), emission spectrum of light-curing unit (LCU), and fluorescence emitted from CDOM which was excited by LCU (**A**); CDOM morphology by TEM observation (**B**); FTIR spectrum of each material (**C**).

The light absorbance of CDOM, emission spectrum of LCU, and fluorescence emitted from the CDOM excited by the LCU (Figure 1 inset) are shown in Figure 1. The CDOM absorbs light mostly in the blue color range, which fully matches the light emitted from the LCU. The CDOM emitted fluorescence with an emission peak near 575 nm.

The light absorbance of CDOM, emission spectrum of LCU, and fluorescence emitted from the CDOM excited by the LCU (Figure 1A inset) are shown in Figure 1. The CDOM absorbs light mostly in the blue color range, which fully matches the light emitted from the LCU. The CDOM emitted fluorescence with an emission peak near 575 nm. The observed CDOM showed a typical morphology at the size of near or less than 10 nm (Figure 1B). FTIR spectra of the used materials (methionine and o-phenylenediamine) and resultant CDOM) are shown in Figure 1C. In the course of hydrothermal synthesis, some bond groups were disappeared and others were newly formed. N-H group in methionine and o-phenylenediamine and S-H group at 2910 cm^−1^ in methionine were not visible in CDOM. C-H group was not changed, C=O group in CDOM indicate the existence of carboxyl group on the surface. Appearance of new group N-S at 1265 cm^−1^ in CDOM may be related with the bonding of CH_3_-detached methionine to -NH_2_ residues of o-phenylenediamine. The formation of N-S group was identified from XPS spectrum of CDOM at 161–166 eV.

**Figure 2:**
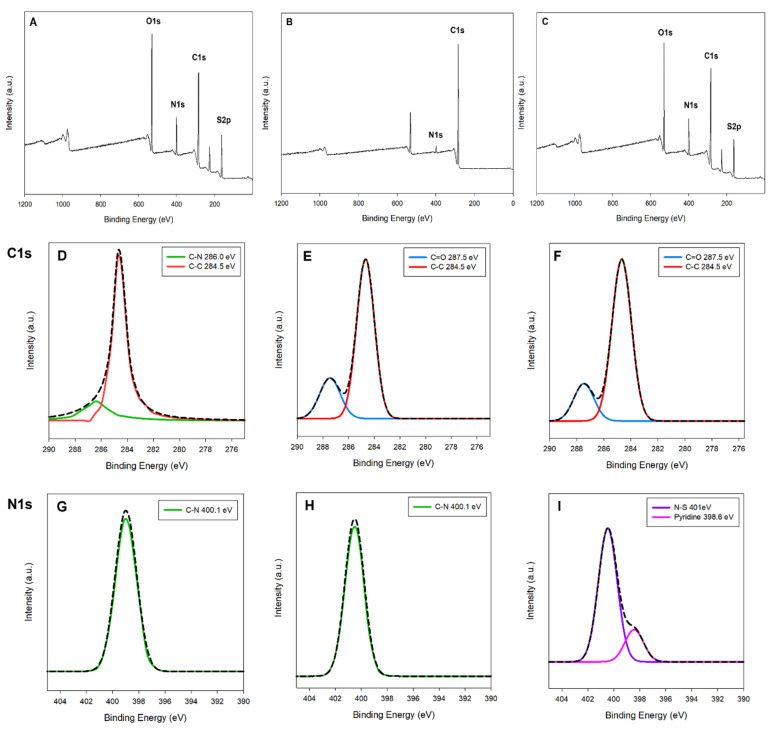

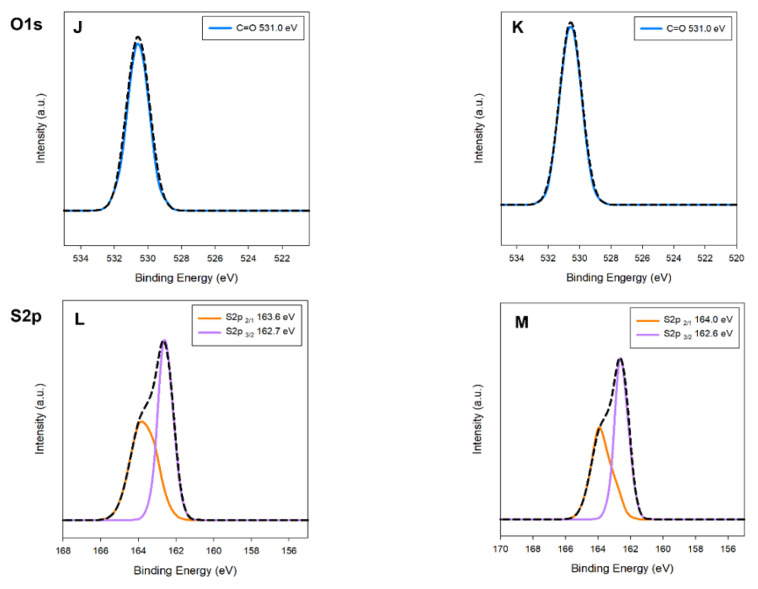
XPS survey spectra of methionine (**A**), o-phenylenediamine (**B**), and CDOM (**C**); C1s high-resolution spectra of methionine (**D**), o-phenylenediamine (**E**), and CDOM (**F**); N1s spectra of methionine (**G**), o-phenylenediamine (**H**), and CDOM (**I**); O1s spectra of methionine (**J**) and CDOM (**K**); S2p spectra of methionine (**L**) and CDOM (**M**).

X-ray Photoelectron Spectroscopy (XPS) analysis of the synthesized CDOM (Carbon Dot from o-Phenylenediamine and Methionine) was performed (Figure 2). The resultant XPS spectra revealed changes in elemental composition and chemical bonding states when compared CDOM to its precursors, o-phenylenediamine and Methionine. Through the survey scanning four typical C, N, O, S peaks were observed (Figure 2A–C). According to the deconvolution based on high-resolution XPS spectra, C1s spectrum of CDOM was observed at 287.5 eV, a peak corresponding to the carbonyl group (C=O) (Figure 2E,F). This indicates that the methionine carboxyl group remained intact even after the synthesis process. In contrast, the C-N bond peak at 286.0 eV (Figure 2D), which was previously observed in the precursor methionine, was not detected in the CDOM spectrum, suggesting that the reaction predominantly occurred at the amino (-NH_2_) residues of o-phenylenediamine. For N1s spectrum, the C-N bond peaks previously identified in the 399–400 eV range for o-phenylenediamine and methionine (Figure 2G,H) were no longer observed in CDOM. Instead, a new N-S bond peak was detected at 401 eV, and a binding energy peak at 398.6 eV, which corresponding to the pyridine-type nitrogen structure, was identified (Figure 2I). These changes demonstrate that the chemical environment of nitrogen atoms within CDOM was restructured into new forms different from those in the precursors. The O1s spectrum showed similar results in both methionine and CDOM (Figure 2J,K), indicating that the methionine carboxyl group was stably preserved after CDOM synthesis. The S2p spectrum analysis revealed particularly significant bonding changes. In the S2p spectrum of methionine (Figure 2L), a peak corresponding to the C-S bond with a spin-orbit splitting distance of approximately 0.9 eV was observed. In contrast, the S2p spectrum of CDOM showed a conversion to an N-S bond form, with the spin-orbit splitting distance increasing to 1.4 eV (Figure 2M). This provides evidence that the sulfur (S) atom formed new chemical bonds with an atom other than carbon (C), and when compared with FTIR results, it appears that N-S bonds were newly generated.

**Figure 3:**
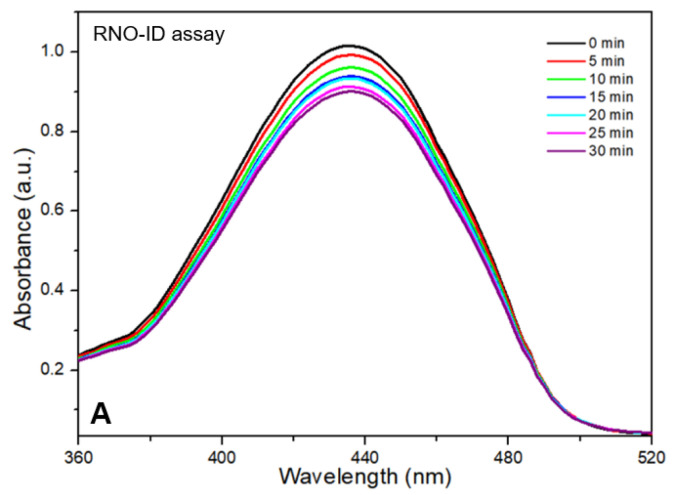

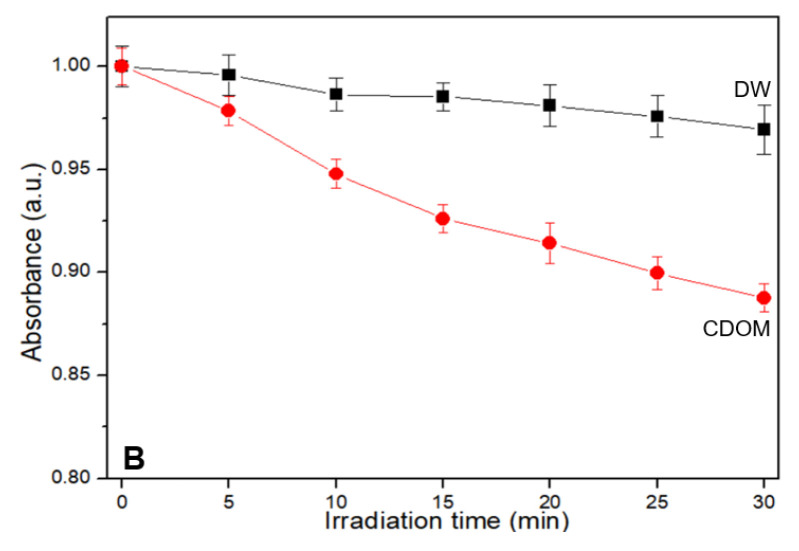
Results of the p-nitrosodimethylaniline-imidazole (RNO-ID) assay for different light irradiation times (**A**). As the light irradiation time increased, the peak intensity obtained from the CDOM-treated assay solution decreased in a close to linear manner (**B**).

The results of the RNO-ID assay are shown in Figure 3. The assay solution absorbed light at 340–520 nm with an absorption peak near 440 nm (Figure 3A). As the irradiation time increased, the peak intensity obtained from the CDOM-treated assay solution decreased almost linearly. After 30 min of light irradiation, the initial peak intensity decreased by approximately 11%, whereas it decreased by 3% in DW without CDOM (Figure 3B).

**Figure 4:**
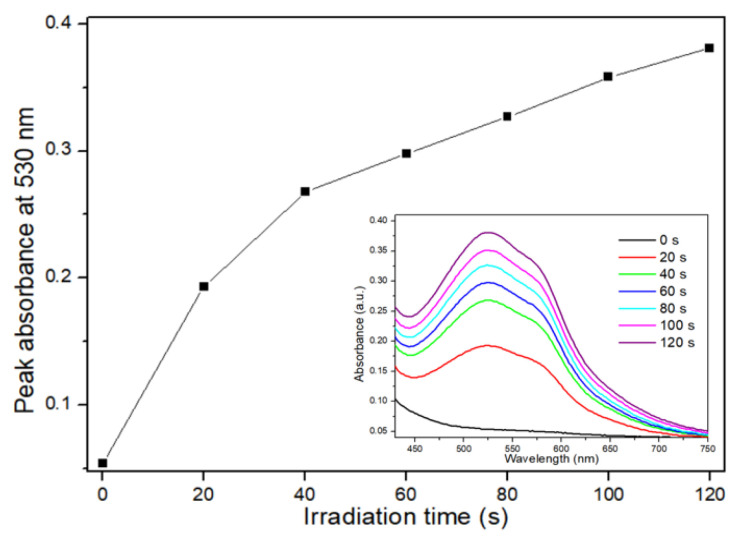
Results of the nitroblue tetrazolium (NBT) assay for different light irradiation times. As the light irradiation time increased, the peak absorbance at 530 nm increased quickly during the first 40 s and then increased in a near to linear manner.

Figure 4 shows the results of the NBT assay. The initial NBT spectrum near the baseline gradually increased at 450–750 nm, with two peaks near 530 and 560 nm, with increasing irradiation time (Figure 4, inset). As the light irradiation time increased, the peak absorbance at 530 nm increased quickly for 40 s and then became nearly linear.

**Figure 5:**
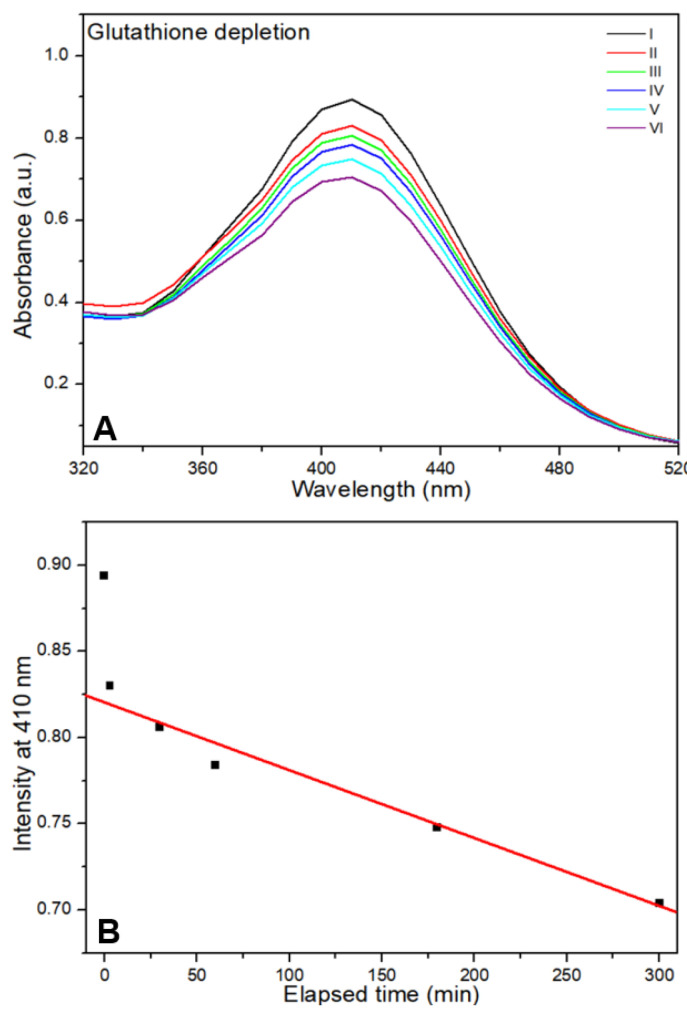
Absorbance change in the glutathione (GSH) assay solution (**A**). During the light irradiation for 3 min, the initial absorbance of the assay solution showed a significant peak intensity decrease near 410 nm. Even after the termination of light irradiation, the decrease continued linearly with time (**B**).

Changes in the absorbance of the GSH assay solution under different conditions are shown in Figure 5. The assay solution absorbs light at wavelengths of <300–520 nm, with an absorption peak near 410 nm (Figure 5A). The initial absorbance of the assay solution showed a significant decrease in the peak intensity near 410 nm during light irradiation for 3 min. Even after the termination of light irradiation, the decrease continued linearly with time (Figure 5B).

**Figure 6:**
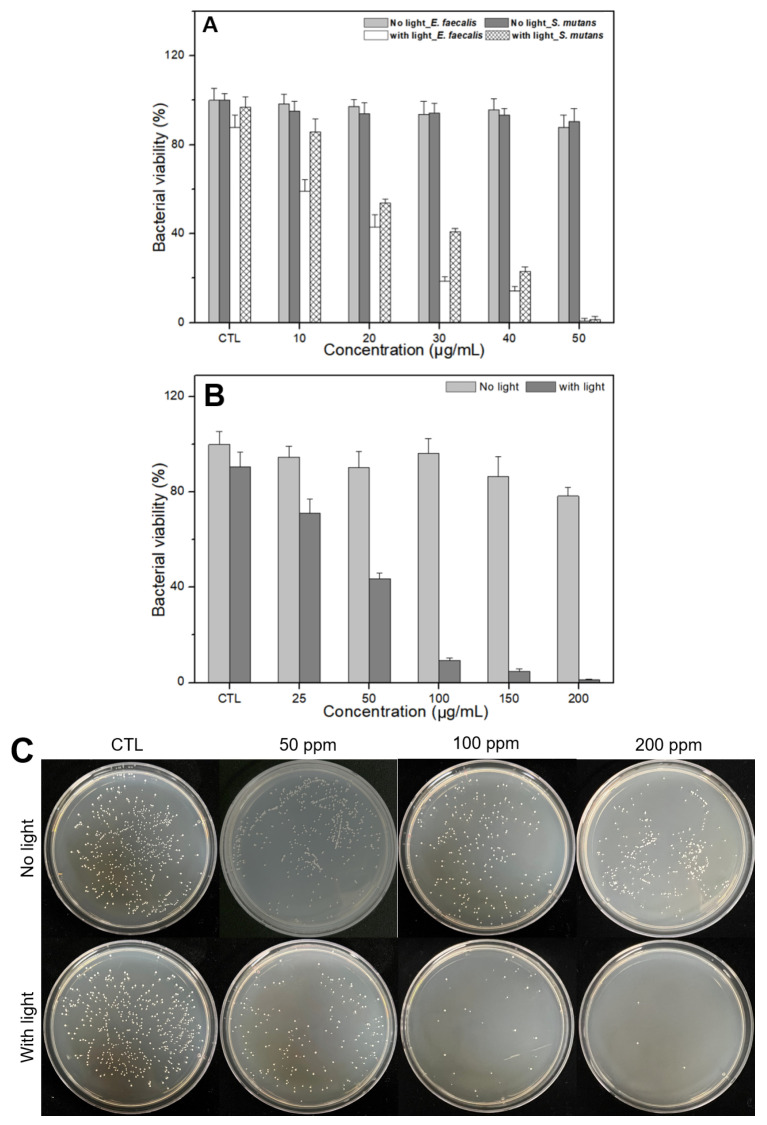
Results of antibacterial tests. *E. faecalis* and *S. mutans* were near totally eliminated with 50 μg/mL of CDOM (**A**) and *C. albicans* were also near totally eliminated with 200 μg/mL of CDOM under light irradiation (**B**,**C**). In the no-light condition, such high elimination was not possible.

The results of the antibacterial tests are shown in Figure 6. According to the tests, *E. faecalis* and *S. mutans* were insignificantly eliminated under the no-light condition, whereas near total elimination was observed under light irradiation with 50 μg/mL of CDOMs (Figure 6A). *C. albicans* were also insignificantly eliminated under the no-light condition at up to 100 μg/mL of CDOM and near to 22% with 200 μg/mL of CDOM, whereas near total elimination was observed under light irradiation with 200 μg/mL of CDOM (Figure 6B). Similar results for colony formation in the dishes are shown in Figure 6C. Light irradiation had a significant impact on the elimination of the two oral bacteria and *C. albicans* compared with the no-light condition (*p* < 0.05).

**Figure 7:**
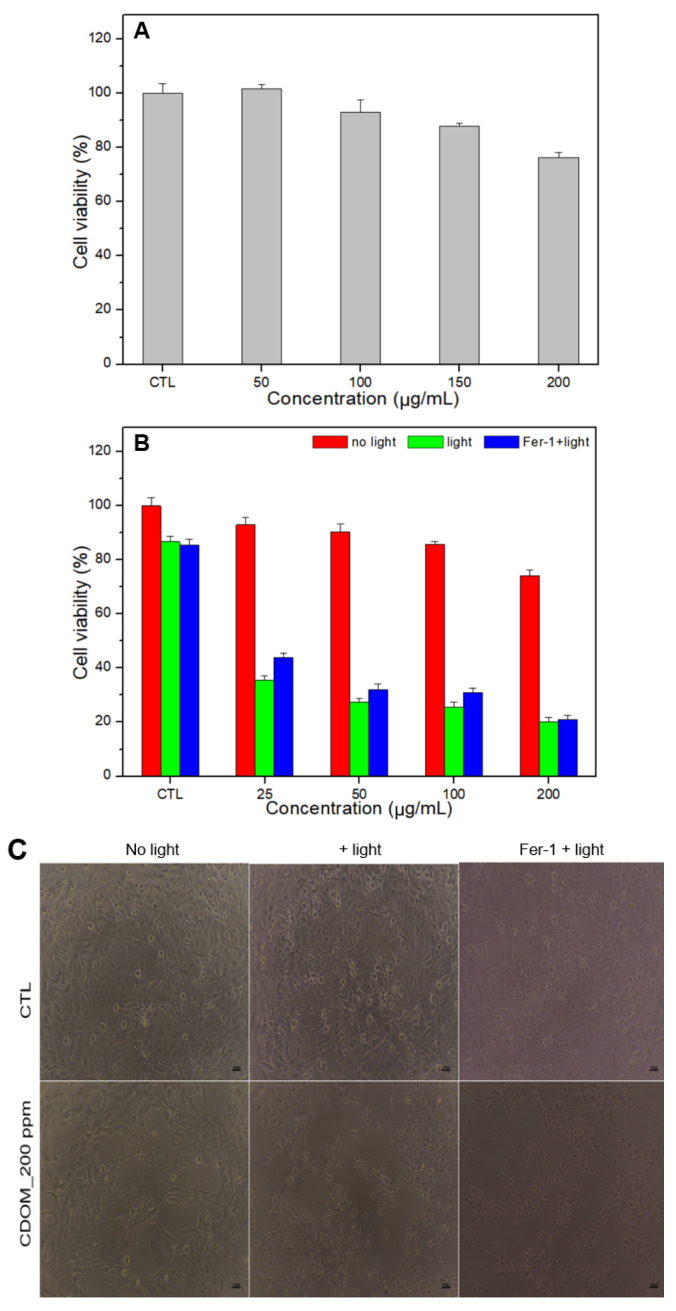
Cell viability of normal (HEK293) and oral cancer cells (HSC3) at different concentrations of CDOM. Normal cells were less damaged at a 200 μg/mL concentration of CDOM (**A**), whereas up to 80% cancer cells were eliminated with no visible live cells under light irradiation. Fer-1 co-treated HSC3 cells showed higher cell viability than the Fer-1-untreated cells (**B**,**C**).

The results of the cell viability tests for normal and cancer cells are shown in Figure 7. CDOM caused less than 10% damage to normal cells (HEK293) with 100 μg/mL of CDOM and approximately 23% damage with 200 μg/mL of CDOM (Figure 7A). For cancer cells (HSC3), the CDOM caused a significant difference in cell viability with light irradiation (*p* < 0.05) compared to the no-light condition. Light-irradiated cells were damaged up to 80% under 200 μg/mL of CDOM while cells in the no-light condition were damaged approximately 25%. Fer-1 co-treated HSC3 cells showed higher cell viability than no Fer-1 treated HSC3 cells (Figure 7B). The optical microscopy images were consistent with the results of the cell viability tests. For 200 μg/mL of CDOM, only light-irradiated cells and Fer-1-treated and light-irradiated cells showed a similar cell state with no visible live cells (Figure 7C).

**Figure 8:**
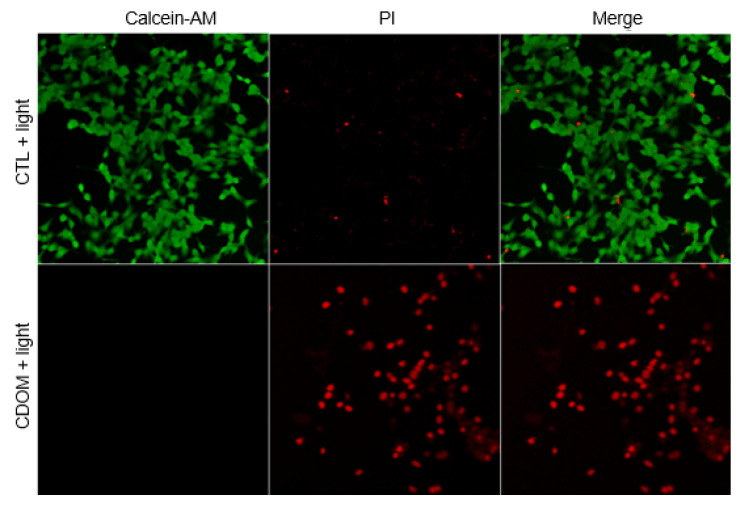
Confocal microscope images of the HSC3 cells after live/dead assay. CDOM-untreated control cells showed green fluorescent spots, whereas CDOM-treated and light-irradiated cells showed many red fluorescent spots owing to PI staining.

Confocal microscopy images of live/dead cell staining after treatment are presented in Figure 8. CDOM-untreated control cells showed green fluorescent spots, whereas CDOM-treated and light-irradiated cells showed many red fluorescent spots owing to PI staining.

**Figure 9:**
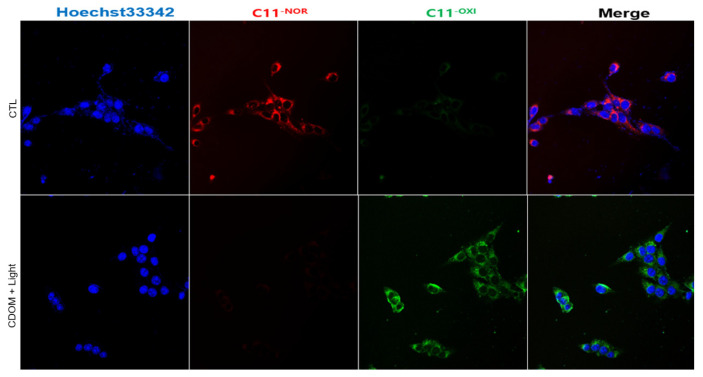
Intracellular lipid peroxidation (LPO) accumulation in HSC3 cells observed using the C11-BODIPY^581/591^ dye. The control cells exhibited red fluorescence (no CDOM, but with light irradiation), whereas cells treated with CDOM and light irradiation exhibited green fluorescence, indicating the accumulation of intracellular LPO.

Intracellular LPO in HSC3 cells was observed using the C11-BODIPY^581/591^ dye (Figure 9). The control cells exhibited red fluorescence (no CDOM, but with light irradiation), whereas cells treated with CDOM and light irradiation exhibited green fluorescence, indicating the oxidation of C11-BODIPY^581/591^ and intracellular LPO.

## 4. Discussion

As the entrance to the digestive system, the oral cavity has complex anatomy, functions, and components. However, owing to the presence of many oral pathogens on the teeth and gingiva, diverse oral diseases can be triggered by these pathogens. Among pathogens that reside in the oral cavity, *S. mutans* is the major contributor to dental caries. *S. mutans* is mostly found on the tooth surface; however, if the bacteria reach the pits or fissures, caries are more likely to occur owing to difficulties in proper treatment in these areas. *E. faecalis* is an opportunistic pathogen that primarily inhabits in the human intestinal tract and oral cavity and is capable of causing severe infections. *E. faecalis* has also been found in reinfected and root canal-treated teeth. According to previous studies, although the conventional agents NaOCl and chlorhexidine can eliminate *E. faecalis,* they are frequently found in treated roots owing to their high viability in harsh environments [19,20]. *C. albicans* is a ubiquitous fungal organism that colonizes the oral cavity in 30–70% of healthy individuals without causing disease [21]. However, in immunocompromised individuals, *C. albicans* infections lead to the development of oral candidiasis. According to a recent meta-analysis, the prevalence of dental caries was high in individuals with *C. albicans* in the oral cavity [22]. In these situations, treating these oral bacteria in one way using a simple approach while the patient is in a chair would be beneficial, but has thus far been challenging for practitioners.

ROS are highly reactive forms of molecular oxygen that exert harmful effects on cells by inducing cellular damage and diseases at high levels. Thus, the generation of large amounts of ROS within a limited time through external intervention is a useful approach to damage bacteria and malignant cells by utilizing oxidative stress. In this study, two different ROS (^1^O_2_ and ^•^O_2_^−^) were identified through assays under light irradiation. Concerning light irradiation, two typical reactions can produce ROS: photodynamic and photocatalytic reactions. In the photodynamic reaction, the photosensitizer (PS) is excited after absorbing light and transfers energy to ground state ^3^O_2_, which then becomes ^1^O_2_ (type II reaction) or transfers electrons to the neighbor oxygen, then other ROS, such as ^•^OH, H_2_O_2_, ^•^O_2_^−^, can be produced (type I reaction). Photocatalytic reaction occurs through interactions between light and the catalyst [23,24]. If the irradiated light is absorbed onto the catalyst, the production of ^•^O_2_^−^ and ^•^OH occurs through the generation of electron-hole pairs and the subsequent chemical reactions by electrons and holes. In these reactions, H_2_O_2_, whether produced during the reaction or previously formed in the tumor microenvironment, contributes to cell damage and oxygen generation within the tumor [25,26]. To date, many PSs have been introduced for the photodynamic therapy. TiO_2_ and g-C_3_N_4_ are well-known photocatalysts. In addition to these traditional PSs and photocatalysts, many CDs have been reported to act as PS or photocatalyst and exhibit excellent antibacterial or antitumor activity [27,28]. The tested CDOM seems to be one of such CDs. To induce photodynamic or photocatalytic reaction, the absorbance of CDs should match the emission spectrum of the incident light. If the absorbance and emission spectrum match, either a photodynamic or photocatalytic reaction may occur depending on the favorable conditions. Finally, the produced ^1^O_2_ degrades RNO, resulting in a decrease in the absorbance of the assay solution. The produced ^•^O_2_^−^ oxidizes NBT and produces insoluble blue formazan, resulting in the absorbance increase in assay solution at 450–650 nm.

GSH is an antioxidant tripeptide that plays a pivotal role in cell protection by reducing oxidative stress. Changes in GSH content were assayed using DTNB (Ellman’s reagent) during and after light irradiation. When GSH reacts with DTNB, it breaks the disulfide bond in DTNB and forms a GSH-TNB adduct and yellow 2-nitro-5-thiobenzoic acid (TNB). Such formed TNB becomes the source of initial absorbance, which has the absorption peak near 412 nm. However, if TNB interacts with formed ROS, it changes to colorless DTNB, then the absorption peak near 412 nm decreases with time, which indicates the depletion of GSH [29,30]. The amino groups on the CDOM surface interacted with GSH, promoting the transfer of electrons from the thiol group (-SH) of GSH to oxygen, thereby creating GSSG. In this study, over time, much depletion occurred during light irradiation and then continually decreased even after the termination of light irradiation. GSH depletion by ROS decreases the cellular activity of GPx and GPx4, which are cellular antioxidants. The formed ROS produce lipid radicals and leads to increased accumulation of lipid peroxides and cell damage (LPO). Excessive LPO leads to another way of cell death (ferroptosis) [31,32]. The oxidation of C11-BODIPY^581/591^ characterizes the formation of cellular oxidative stress and subsequent cell death, which is manifested by green fluorescence in treated cells [33,34].

To evaluate whether CDOM can eliminate both oral bacteria and oral cancer in vitro, bacterial viability tests were performed. *E. faecalis* and *S. mutans* were near totally eliminated under light irradiation with 50 μg/mL concentration. *C. albicans* was also near totally eliminated under light irradiation with 200 μg/mL concentration. However, this elimination is not possible in the absence of light irradiation. Similar results were observed in cancer cells in vitro. Cell viability tests were performed using HSC3 cells in the absence or presence of Fer-1. CDOM eliminated approximately 25% of HSC3 cells without light irradiation, yet at 200 μg/mL concentration, it eliminated 80% cells with light irradiation. An increase in cell viability in the presence of Fer-1 indicates that Fer-1 is a ferroptosis inhibitor [35,36,37] which means CDOM is involved in ferroptosis as a cell-death mechanism. Although the CDOM induced high levels of cancer cell death, it showed insignificant toxicity in normal cells. The cell viability was approximately 93% and 74% for 100 μg/mL and 200 μg/mL concentration, respectively. Thus, on using an LCU, CDOM can near totally eliminate both oral bacteria and oral cancer cells at concentrations of not more than 50 or 200 μg/mL by ROS-induced (apoptosis) or ROS-initiated GSH depletion-induced (ferroptosis) intracellular LPO through the photoexcitation of CDOM.

## 5. Conclusions

Within the limitations of this in vitro study, we found that the synthesized CDOM could near totally eliminate both oral bacteria (*C. albicans*, *E. faecalis*, *S. mutans*) and oral cancer cells (HSC3) at 50 and 200 μg/mL concentration, respectively, with an aid of a dental LCU. The produced ROS (^1^O_2_ and ^•^O_2_^−^) or intracellular LPO driven by ROS-initiated GSH depletion is believed to be part of the lethal mechanism for this in vitro antibacterial and antitumor therapy. The dental LCU worked effectively as a light source for the production of lethal ROS through the photoexcitation of CDOM. To extend the potential applicability of the present modality, the extended and deepened investigations with in vivo study are asked for the future dental clinical workflows.

## Data Availability

Data are contained within the article.
